# Phenolic Compounds with Antioxidant Properties from Canola Meal Extracts Inhibit Adipogenesis

**DOI:** 10.3390/ijms21010001

**Published:** 2019-12-18

**Authors:** Saira Hussain, Ata Ur Rehman, David J. Luckett, Christopher L. Blanchard, Hassan K. Obied, Padraig Strappe

**Affiliations:** 1 ARC Industrial Transformation Training Centre for Functional Grains & Graham Centre for Agricultural Innovation, Boorooma Street, Wagga Wagga, NSW 2650, Australia; rehmanata@hotmail.com (A.U.R.); cblanchard@csu.edu.au (C.L.B.); 2 Graham Centre (an alliance between Charles Sturt University and NSW Department of Primary Industries), Boorooma Street, Wagga Wagga, NSW 2678, Australia; djluckett@gmail.com; 3 School of Biomedical Sciences, Charles Sturt University, Boorooma Street, Wagga Wagga, NSW 2678, Australia; obiedhk@gmail.com; 4 School of Medical and Applied Sciences, Central Queensland University, Rockhampton, Qld 4702, Australia; p.strappe@cqu.edu.au

**Keywords:** *Brassica napus*, rapeseed, polyphenols, adipogenesis, pancreatic lipase, antioxidant

## Abstract

The extraction of phenolic compounds from canola meal produces functional health products and renders the canola meal a more digestible animal feed. The extracted phenolics may have novel bioactivity worth investigation. In this study, several solvents were evaluated for their ability to extract phenolic compounds from canola meal: water (WE) and various 80% organic solvent/water mixtures of methanol (ME), acetone (AE), ethanol (EE), butanol (BE), chloroform (CE) and hexane (HE). The in vitro antioxidant and anti-obesity properties of various extracts were investigated. Anti-obesity properties were studied using adipogenic differentiation inhibition of a murine mesenchymal stem cell line (C3H10T1/2) and a pancreatic lipase inhibition assay. AE, ME, and BE showed significant (*p <* 0.05) adipogenesis and pancreatic lipase inhibitory activities and may have more pharmacological properties. AE down-regulated the gene expression of the major adipogenic transcription factor, peroxisome proliferator-activated receptor gamma (PPARγ), correlating to phenolic content in a dose-dependent manner. The chemical characterization of AE revealed the presence of sinapic acid, ferulic acid, and kaempferol derivatives as main bioactive phenols.

## 1. Introduction

Canola (*Brassica napus* L., rapeseed, oilseed rape) is used as a medicinal food in Middle Asia, North Africa, and West Europe. There is also evidence of its use by early Australian and New Zealand settlers as well as the indigenous population [1]. Large amounts of protein-rich canola meal are generated globally during the extraction of canola seed oil. Over 70 million tonnes of canola/oilseed rape is produced per year, resulting in over 40 million tonnes of canola meal. The high phenolic content of canola meal renders it less appealing as an animal feed. On the other hand, plant phenols are attracting increasing attention as multipotent antioxidant molecules that can be used to generate high-value nutraceutical products. The extraction of canola meal phenols is an appealing high-tech solution to generate antioxidant-rich extracts for the food and pharmaceutical industries while enhancing the value of the remaining meal by increasing its digestibility [2].

Polyphenols are a major component of the bioactive molecules isolated from plant extracts and have been associated with their ability to modify a range of diseases, including cardiovascular disease [3,4]. Inflammation which underlies chronic vascular diseases such as atherosclerosis has been shown to be targeted by different types of polyphenols such as lignans, phenolic acids and a variety of flavonoids. At the cellular level, the mode action of polyphenols as anti-inflammatory mediators [5] includes antioxidant activity [6], regulating cell signaling associated with downregulation of pro-inflammatory cytokine expression [7] and reducing endothelial cell dysfunction [8]. A number of population studies have linked dietary flavonoid intake with reduced cardiovascular disease [9] with a possible stronger association with a beneficial effect with flavonols and flavones. More recently a meta-analysis of dietary flavonoid and lignan intake and mortality found that flavonoids were associated with a decreased risk of chronic vascular disease mortality [10].

It is well understood that phenolic extracts such as those found in canola often demonstrate in vitro antioxidant activity [11,12]. However, antioxidant activity does not always correlate with clear health properties. A more useful approach to characterizing plant biophenols is by assessing their bioactivity in systems that mimic physiological conditions. As animal and clinical studies have limitations (e.g., high cost and low throughput), in vitro bioassays to screen for pharmacological activities are a more useful alternative especially at early stages of biological screening.

Earlier work has pointed to the importance of canola-derived by-products as potential bioactive compounds. Canola meal proteins and hydrolysates have both been shown to possess pharmacological properties [13]. Canola meal protein isolates and enzymatic hydrolysates have also been shown to have anti-obesity properties in vitro [14]. However, the anti-obesity properties of canola meal biophenols have not yet been investigated.

Obesity is increasing worldwide [15], due to lifestyle changes, less physical exercise, and unhealthy eating habits [16]. A hallmark of obesity is the accumulation of fat tissue as a result of prolonged increase in energy intake compared to output, and it is a major risk factor in many human diseases including diabetes mellitus, cancer, atherosclerosis, and hypertension [17]. Oxidative stress plays a significant role in the pathology of obesity [18]. Very few medications are approved as anti-obesity agents. Yet, their cost, efficacy and safety are largely disappointing. Thus, there is a stressing need to find more affordable, effective and safer alternatives [19]. Plant phenols, indirectly as well-known antioxidants and directly through their anti-obesity activities [20], have been shown to be an unrivaled resource of newer anti-obesity agents.

Adipocytes are specialized insulin-sensitive cells that store fat and secrete hormones. Most of the fats stored in adipocytes are in the form of triglycerides. Adipocytes originate from progenitor cells (preadipocytes), and the process of differentiation to adipocytes is termed adipogenesis [21]. At the cellular level, obesity is associated with hypertrophy of adipocytes and the production of new adipocytes from precursor cells. A potential anti-obesogenic approach can be through the inhibition of pre-adipocytes differentiation to mature cells, hence reducing adipocyte numbers. Inhibition of the adipogenic differentiation pathway is commonly assessed using the mouse preadipocyte “3T3 L1” clonal cell line which has been used to determine the impact of compounds regulating adipogenesis [22]. An alternative cell line, “C3H10T1/2”, is a multipotent embryonic mesenchymal stem cell line that can be differentiated into adipocytes, and has been previously used to demonstrate anti-adipogenic properties [23].

During differentiation, the transcription factor peroxisome proliferators-activated receptor gamma (PPARγ) acts as a master regulator of adipogenesis [14]. PPARgamma (PPARγ), a major regulator of adipogenesis, is a nuclear receptor and is a part of the PPARs family. It accumulates particularly in adipose tissues to regulate the expression of genes with their essential role to differentiate [24]. Growth of triglycerides (TG) in mature adipocyte is triggered when adipogenic transcriptional factors such as PPARγ/CCAT/enhancer-binding protein (C/EBP) are expressed [24]. It leads to a pathway to regulate fatty acid synthase (FAS) and acetyl-CoA carboxylase (ACC) [25]. PPARγ can detect lipase activity [26]. Therefore, bioactivity of canola extracts can also be assessed in relation to their capacity to inhibit pancreatic lipase exclusively as the concentration that inhibits 50% (IC50) of pancreatic lipase (PL) activity in contrast to standard Orlistat.

Sinapic acid [27], and hydroxycinnamic acids [28] are known for the lipolysis and their role in anti-adipogenesis. Polyphenols including quercetin, resveratrol, apigenin, and myricetin are known to bind with particular residues (Phe264, His266, Ile281, Cys285, and Met348) of the PPARγ receptor to establish anti-adipogenicity during the early stages of the differentiation [29]. For example, ethanol extracts of bamboo stems have been shown to play a vital role in the downregulation of differentiation markers C/EBPβ, PPARγ, and FABP4 (fatty acid-binding protein) [30]. Likewise, extracts of *Mangifera indica* L. leaf have also been shown to be effective in reducing the effect of PPARγ suggesting their potential use in obesity management [31]. Furthermore, polyphenol extracts of purple maize (Zea mays L) and purple silk corn extracts have been shown to have activities such as anti-inflammatory,anti-adipogenic,anti-diabetic and induction of lipolysis [32,33]. Moreover, pomegranate juice has also been shown to down-regulate adipogenic genes and lipase [34]. Specific compounds identified include quercetin, luteolin, vanillic acid and protocatechuic acid, rutin [33], ellagic acid and punicalagin [34].

Pancreatic lipase is a key enzyme secreted from the pancreas and plays a major role in the hydrolysis of 50%–70% of dietary triglycerides to monoacylglycerides and free fatty acids before absorption by enterocytes [20]. Therefore, inhibition of this enzyme may result in lower fat absorption. In this study, various solvent mixtures were used to recover canola meal phenols and study their composition and bioactivities. The phenolic composition of various extracts from canola meal, their in vitro antioxidant and anti-obesity activities were investigated.

## 2. Results

Canola meal was extracted with water (WE) and various 80% organic solvent/water mixtures of methanol (ME), acetone (AE), ethanol (EE), butanol (BE), chloroform (CE) and hexane (HE). The canola meal extracts CME were chemically characterized and screened for their anti-obesity properties.

### 2.1. Phenolic Composition of Canola Meal Extracts

**Figure 1:**
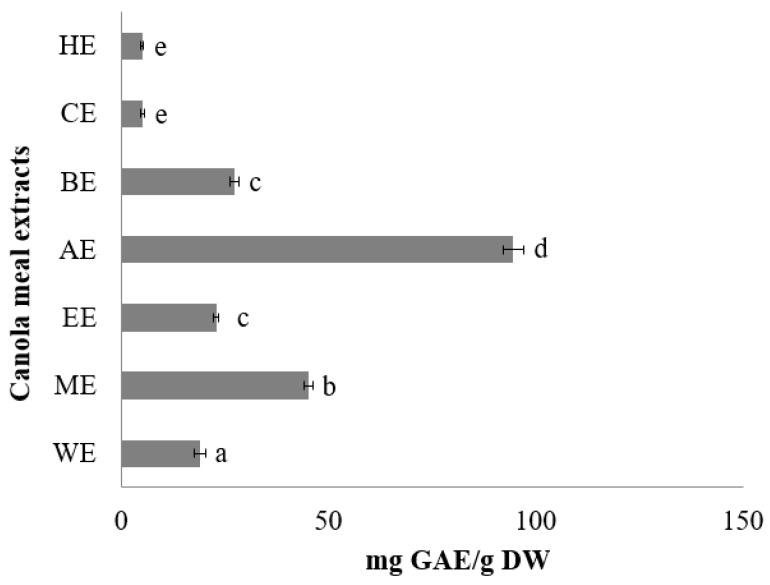
Total phenol content of canola meal extracts (CMEs) expressed as mg GAE/g extractable matter. WE: water extract, ME: methanol extract, EE: ethanol extract, AE: acetone extract, BE: butanol extract, CE: chloroform extract, HE: hexane extract. Bars with different letters have means that are significantly different (*p < 0.05*).

Gallic acid was used to produce a calibration curve by linear regression for the analysis of total phenolic content (TPC) from canola meal extracts (*R^2^* = 0.9998). The TPC of various extracts in decreasing order was as follows: AE > BE > ME > EE > WE > CE > HE (Figure 1).

**Figure 2:**
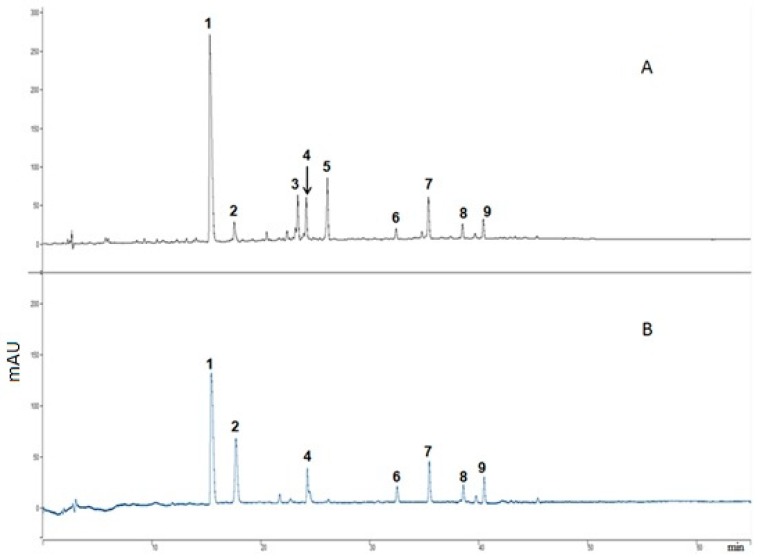
On-line HPLC-antioxidant analyses of acetone extract. Eluting time in minutes (x-axis) and absorbance in mAU (Y-axis). (**A**) Chromatogram at 280 nm (**B**) ABTS scavenging detection at 414 nm: Compounds identified were (1) sinapine, (2) feruloyl choline (4-0-8’) guiacyl, (3) unknown, (4) feruloyl choline (5-8’) guaiacyl, (5) kaempherol-sinapoyl-trihexoside, (6) *trans*-sinapic acid, (7) feruloyl choline (4-O-8’) guaiacyl-di-sinapoyl, (8) disinapoyl dihexoside, and (9) disinapoyl hexoside.

Various extracts were chemically characterized by HPLC-DAD-MS/MS using commercially available reference standards and spectral data from previous literature [2]. Different extracts showed different phenolic profile [35] that reflects the relative polarity of various extracting solvents. In accord with total phenolic content, the acetone extract AE showed the largest number of peaks and the highest recovery (peak height). Most peaks were of intermediate polarity, eluting between 15–40 min in the 60-minute long chromatograms (Figure 2). In alcoholic and acetone extracts, the major peak observed was sinapine (Peak 1). The major peak observed in water, chloroform, and hexane extracts was feruloyl choline (4-O-8’) guaiacyl-di-sinapoyl (Peak 7).

Compounds present in AE were characterized by HPLC-DAD with online detection of free radical scavenging activity of eluting compounds using an ABTS scavenging assay, as shown in Figure 2, Table 1. Peaks 1–9 in Figure 2 are the main contributors to the antioxidant activity of the extract (showing peaks in the ABTS chromatogram).

### 2.2. Canola Meal Extracts Inhibit Intracellular Lipid Accumulation

**Figure 3:**
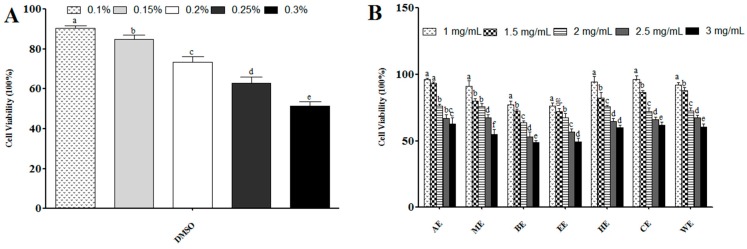
Determination of cell viability using the CellTiter 96^®^ AQueous non-radioactive cell proliferation assay, and adipogenic differentiation inhibition by canola meal extracts (CMEs). (**A**) The effect of DMSO on C3H10T1/2 cell viability. The effect of DMSO concentration (0.1%, 0.15%, 0.2%, and 0.3%) on cell viability was assessed using the CellTiter 96^®^ AQueous non-radioactive cell proliferation assay. Results are expressed as mean ± standard deviation (*n* = 3). Bars with different letters have mean values that are significantly different (*p <* 0.05) at different concentrations. (**B**) The effect of canola meal extracts on C3H10T1/2 cell (CME) cell viability. Viability was assessed using the CellTiter 96^®^ AQueous non-radioactive cell proliferation assay at different concentrations (1, 1.5, 2, 2.5, and 3mg/L) of CMEs. Results are expressed as mean ± standard deviation (*n* = 3). Bars with different letters have mean values that are significantly different (*p <* 0.05) at the same concentration.

Cell viability was determined by the CellTiter 96^®^ AQueous non-radioactive cell proliferation assay according to the manufacturer’s protocol [14]. The CellTiter 96^®^ AQueous assay constitutes of a solution of a novel [3-(4,5-dimethylthiazol-2-yl)-5-(3-carboxymethoxyphenyl)-2-(4-sulfophenyl)-2H-tetrazolium salt (MTS) and an electron coupling reagent (phenazine methosulfate, PMS). MTS is bio-reduced by cells into a formazan product that is soluble in tissue culture medium. Untreated cells served as a control and were assumed to be 100% viable. To assess the effect of DMSO on the viability, cells were incubated with various concentrations of DMSO (0.05%–0.3%) in cell culture medium, DMEM, and the proportion of viable cells was determined (Figure 3A). The toxicity of various extracts on cells was examined in the range 1–3 mg/mL (Figure 3B). 

**Figure 4:**
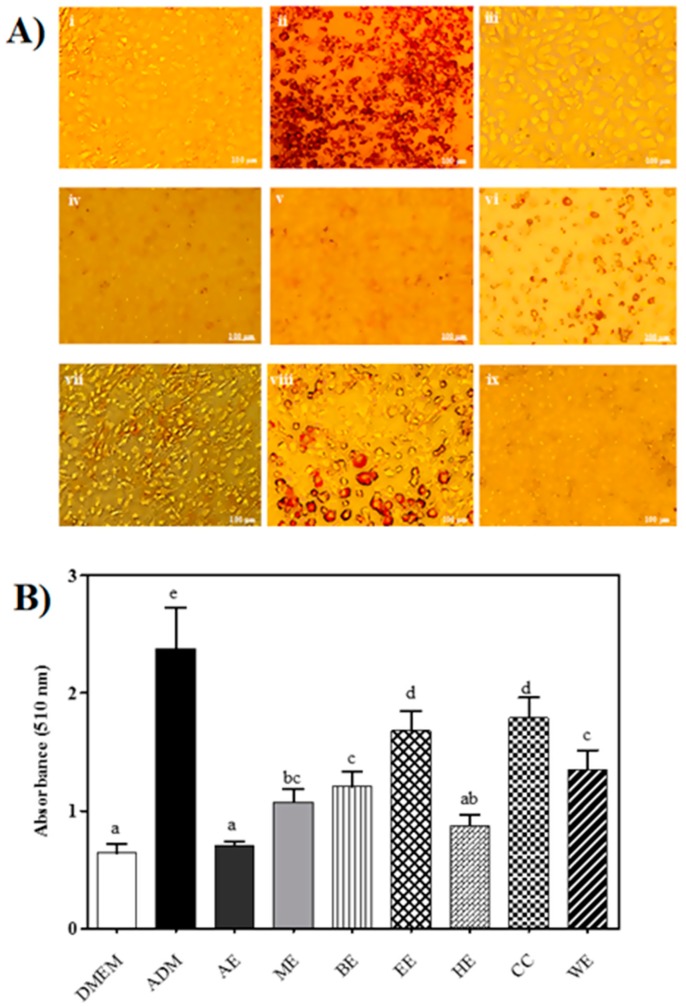
Adipogenic differentiation inhibition by canola meal extracts (CME’s). (**A**) Qualitative assessment of the effect of CMEs on adipogenic differentiation visualized at 10 µm: (i) undifferentiated cells—negative control; (ii) differentiated cells—positive control; differentiated cells with CMEs (iii) acetone (AE), (iv) methanol (ME), (v) butanol (BE), (vi) ethanol (EE), (vii) hexane (HE), (viii) chloroform (CE), and (ix) water (WE). (**B**) Quantitative analysis of adipogenesis inhibition. Oil red staining measured at 510 nm was used to assess the level of adipogenesis in differentiated mesenchymal stem (CH310T1/2) cells incubated with various CMEs. Results are expressed as means ± standard deviation (*n* = 3). Bars with different letters have means that are significantly different (*p <* 0.05).

The in vitro anti-adipogenic effect of CME was examined with 1.5 mg/mL of EE and BE, and 2 mg/mL of AE, ME, HE, CH, and WE in the presence of adipogenic differentiation media (ADM) for seven days. Cells of C3H10T1/2 were then stained with Oil Red O stain (Figure 4A) and visualized by light microscopy and quantified by measuring absorbance at 510 nm (Figure 4B). Fat globules appeared as red granules, as shown in Figure 4A, demonstrating differentiation into adipocytes [36]. Following staining with Oil Red O, a clear distinction can be seen in undifferentiated cells (Figure 4A, panel i) and differentiated cells (Figure 4A, panel ii).

All extracts inhibited stem cell differentiation to some extent. The cells treated with AE showed the highest level of inhibition (*p <* 0.05). In general, we can categorize the CME into the following clusters: strong inhibitors (AE and HE), moderate inhibitors (ME, BE, and WE), and mild inhibitors (EE and CE). Accordingly, large lipid vacuoles were clearly noticeable in EE- and CE-treated cells, showing minimal inhibition had occurred (Figure 4A, panels vi,viii).

Excess storage of lipid as adipose tissue has been identified as a risk factor for the development of many diseases [37]. Although canola meal has never been studied for its anti-adipogenic potential, many naturally-occurring flavonoids, phenolic acids, and lignans have demonstrated a capacity to inhibit the lipid droplet deposition in the adipose tissue [38].

### 2.3. Canola Meal Extract Mediated Reductions in Lipid Accumulation are Correlated with a Reduction in PPARγ Expression

**Figure 5:**
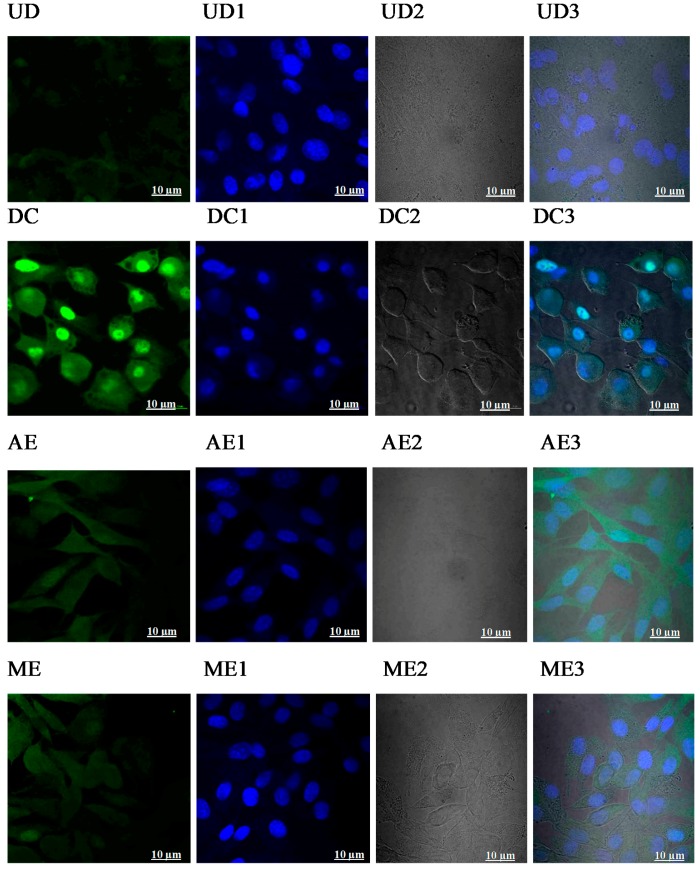

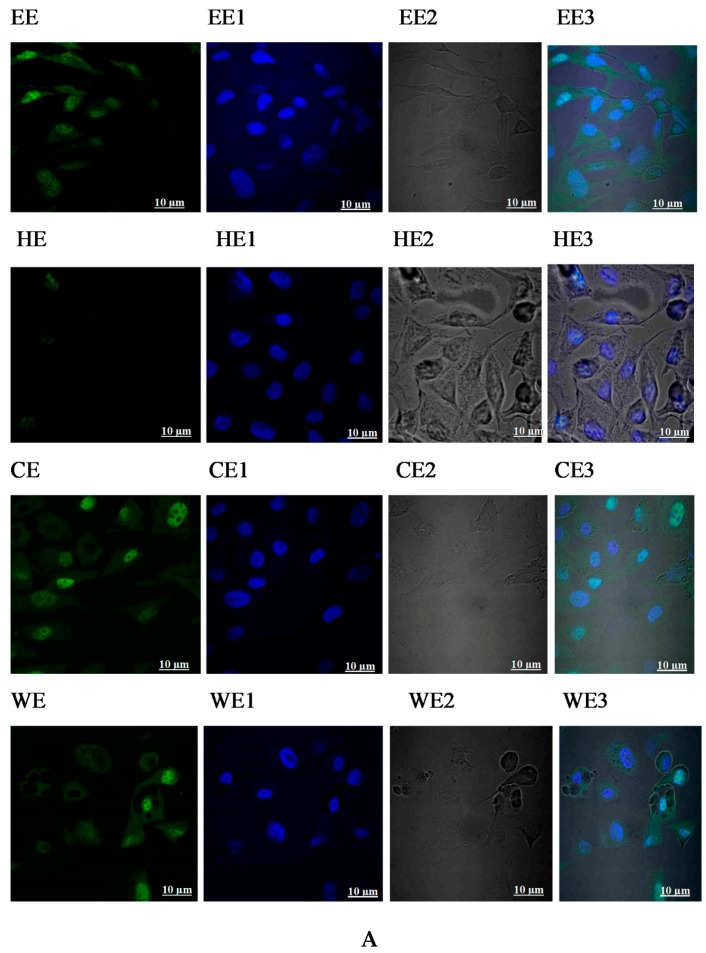

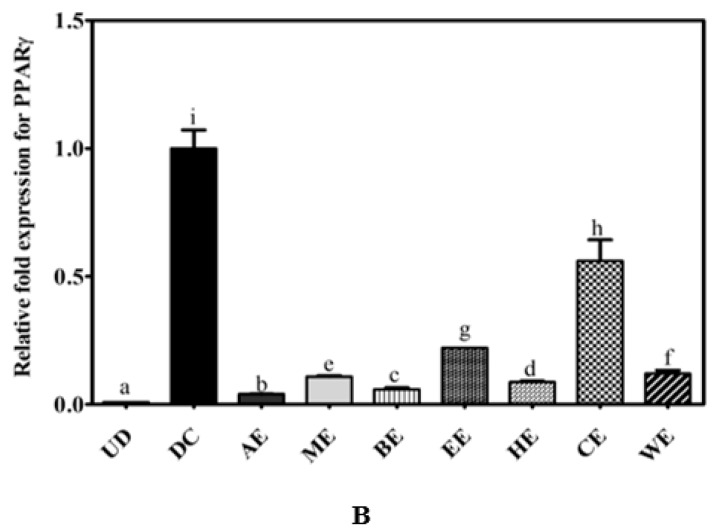
(**A**) Immunofluorescence staining of PPARγ in CME treated mesenchymal stem cells (CH310T1/2) visualized at 10 µm: (**UN**) undifferentiated cells—negative control; (**DC**) differentiated cells—positive control. Differentiated cells treated with CMEs: Acetone extract (**AE**); methanol extract (**ME**); butanol extract (**BE**); ethanol extract (**EE**); hexane extract (**HE**); chloroform extract (**CE**); and water extract (**WE**). Captured images with no number labels are cells stained with fluorescein isothiocyanate (FITC). Images labeled with “1” show cells stained with 4’,6-diamidino-2-phenylindole (DAPI); images labeled with “2” show the normal view of cells, and images labeled with “3” are merged images of FITC stained cells, DAPI stained cells, and normal images, respectively. (**B**) Quantitative analysis of PPARγ gene expression in C3H10T1/2 cells treated with CMEs. Results are expressed as means ± standard deviation (*n* = 3). Bars with different letters have mean values that are significantly different (*p <* 0.05).

To determine the effects of CMEs on PPARγ expression, PPARγ immunostaining of cells undergoing differentiation was undertaken (Figure 5A,B). The typical nuclear localization of PPARγ was seen in differentiated cells (Figure 5B, panel DC). No nuclear PPARγ staining was observed for either AE- or BE-treated cells, and a low level of staining was observed in HE-treated cells. BE appeared to inhibit adipogenic differentiation at the lower dose of 1.5 mg/mL. These results indicate that CMEs appear to differ in their ability to inhibit adipogenesis to different extents. The level of PPARγ gene expression in the cells treated with CMEs was determined using qRT-PCR (Figure 5B). Cells incubated in culture media alone acted as a negative control and untreated fully-differentiated cells were used as a positive control. The expression level of PPARγ in the C3H10T1/2 cells treated with AE exhibited a significant decrease (*p <* 0.05) compared to the positive control. BE treated cells also showed a lower expression of PPARγ, and a reducing trend was observed as follows: HE < ME < WE < EE < CE. PPAR*γ* gene expression levels followed the same trend seen with Oil Red O staining (Figure 4A) and PPAR*γ* immunofluorescence staining (Figure 5A).

Previous studies have shown that reduced PPAR*γ* gene expression correlates with inhibition of adipogenesis in 3T3-L1 adipocyte cells treated with extracts of cranberries and onion peel [22,39]. Also, canola proteins and their hydrolysates have been shown to reduce the expression of genes coding for PPAR*γ* proteins in C3H10T1/2 cells [14].

### 2.4. Inhibition of Pancreatic Lipase Activity

**Figure 6:**
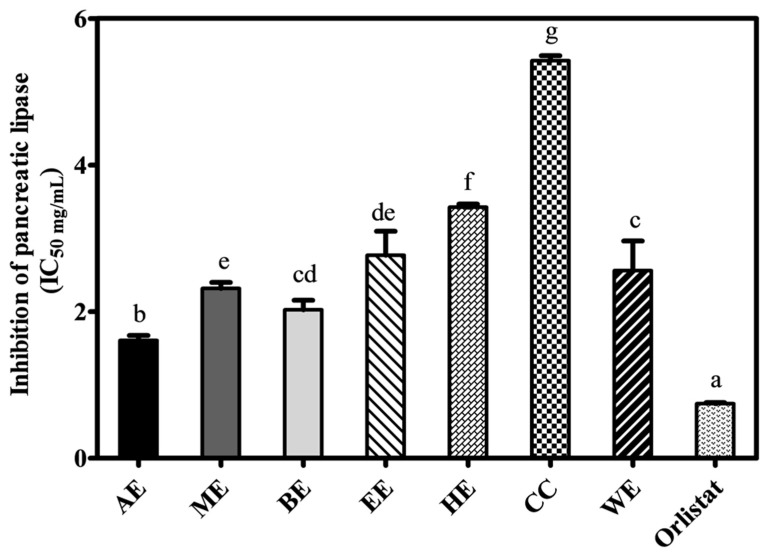
Inhibitory effect of CME (IC_50_) on pancreatic lipase inhibition: Inhibition concentrations at 50% (IC_50_) values of pancreatic lipase (x-axis) activity of Orlistat, AE, ME, BE, EE, HE. CE, WE (y-axis). Results are expressed as means ± standard deviation (*n* = 3). Bars with different letters have mean values that are significantly different (*p* < 0.05).

The IC_50_ values for pancreatic lipase (PL) inhibition of the seven CME are shown in Figure 6. All extracts demonstrated some level of inhibition towards PL with AE showing the highest (1.60 ± 0.06 mg/mL), and the CE the lowest (5.42 ± 0.07 mg/mL) (*p <* 0.05) levels of inhibition. BE also showed a high level of PL inhibition. From these results, we conclude that the phenolic compounds in AE and BE are active in both the inhibition of lipid accumulation in adipocytes and lipase enzymatic activity. Several plant extracts have been shown to have PL inhibitory effects [40].

## 3. Discussion

Acetone showed superior phenol extraction abilities (287.7 ± 16.3 mg GAE/g extractable matter), which has been observed before in other plant extracts [41]. Butanol and methanol extracts showed high levels of extraction, without a significant difference in their abilities to extract canola phenolic compounds. Although ethanol has an intermediate polarity between methanol and butanol, ethanol extracts recovered significantly less phenols. Highly-polar (water) and non-polar (chloroform and hexane) solvents showed the least potential to extract canola phenols. Phenolic profiling of all extracts was undertaken [35]; however, only the chemical characterization of the extract with the highest phenol content (AE) is discussed here. Alcohol and acetone extracts showed the largest number of peaks.

Interestingly, water extracts were comparable to hexane and chloroform extracts in their ability to extract phenolic compounds as these extracts contained almost the same number of peaks. However, water extracts appeared to have higher recovery. The data gathered from UV–vis spectra, ABTS scavenging activity, and relative retention times were compared with reference standards and literature data to characterize the chemical composition of CMEs as described previously [2]. The online-ABTS HPLC analysis showed that, generally, most detected compounds demonstrate good ABTS radical scavenging activity. A few exceptions can be observed, such as Peak 5 (kaempferol-sinapoyl-trihexoside), which had no ABTS scavenging activity. Meanwhile, Peak 2 (feruloyl choline guiacyl) was the sixth most abundant component in AE (Figure 2A), yet it appeared as the second highest peak in the online ABTS radical scavenging trace (Figure 2B), reflecting a strong free radical scavenging activity that is not proportional to its relative concentration.

DMSO (0.2%) solution was selected as the solvent that achieved a complete dissolution of extracts and maintained approximately 70% cell viability in accord with previous studies [42]. The anti-adipogenic effects of samples at these concentrations has been shown not to be due to the effect of DMSO [43]. All extracts demonstrated more than 70% viability at ≤ 2 mg/mL, apart from BE and EE which showed similar viability at ≤ 1.5 mg/mL. Therefore, 1.5 mg/mL of the ethanol and butanol extracts, and 2 mg/mL of all other extracts were used for cell culture experiments (Figure 3B).

All extracts demonstrated significant inhibition of stem cell differentiation. The cells treated with AE showed the highest inhibition (*p <* 0.05). In general, we can categorize the CME into the following clusters: strong inhibitors (AE and HE), moderate inhibitors (ME, BE, and WE), and mild inhibitors (EE and CE). Accordingly, large lipid vacuoles were clearly noticeable in EE- and CE-treated cells showing minimal inhibition had occurred (Figure 4A, panels vi,viii).

The inhibition of PL has been identified as a potential target for the treatment of obesity. So far, many plants have been examined for their PL inhibitory potential [40]. All CMEs showed some PL inhibitory potential (Figure 6). The most potent inhibitor was AE followed by alcoholic extracts, while the least potent were HE and CE. Lipase inhibition correlated well with the degree of adipogenic inhibition measured by Oil Red O staining (Figure 4A).

## 4. Materials and Methods

### 4.1. Materials

Chemicals used included HPLC-grade methanol from Thermo Fisher Scientific (Reagent Lane Fair Lawn, NJ, USA), anhydrous acetonitrile from UNICHROME (Sydney, Australia), sodium carbonate, ferric chloride, Folin–Ciocalteu reagent, 2,2′-azinobis (3-ethylbenzothiazoline-6-sulfonic acid) diammonium salt (ABTS), 2-4-6-tris(2-pyridyl)-s-triazine (TPTZ), potassium persulphate, gallic acid, ferulic acid, sinapic acid, formic acid and membrane nylon filters (0.22 μm), from Sigma Aldrich (Sydney, Australia). Kaempferol was obtained from Extrasynthese (Genay, France). Chemicals including orlistat, 4-methylumbelliferyl oleate (4-MU oleate), tris hydrochloride (Tris-HCl), calcium chloride (CaCl_2_), and sodium citrate, sodium chloride (NaCl) were obtained from Sigma-Aldrich (Sydney, Australia). Pancreatic lipase (PL, porcine) was purchased from Roche Diagnostic (Melbourne, Victoria, Australia).

The murine embryonic fibroblast mesenchymal stem cell line (C3H10T1/2) was purchased from the American Type Culture Collection (Rockville, MD, USA). The CellTitre® AQueous non-radioactive cell proliferation assay kit and GoTaq Green 2× master mix were purchased from Promega (Fitchburg, WI, USA); Aurum™ total RNA kit and iScript™ advance cDNA synthesis kits were obtained from Bio-Rad Laboratories (Hercules, CA, USA); and RT-PCR grade water was purchased from Life Technologies (Scoresby Victoria, Australia).

Cells were cultured in Dulbecco’s modified Eagle’s medium (DMEM) plus 10% Fetal Bovine Serum (FBS), and 1% penicillin and streptomycin, and passaged using a 0.25% trypsin-EDTA solution. Dexamethasone, penicillin/streptomycin, L-glutamine, 3% para-formaldehyde (PFA), and phosphate-buffered saline (PBS) were purchased from Sigma-Aldrich Chemicals (St. Louis, MI, USA).

### 4.2. Recovery of Canola Meal Biophenols

Canola meal was extracted with water and a range of aqueous 80% solvents, namely, acetone, methanol, butanol, ethanol, hexane, and chloroform. Canola meal phenols are essentially hydrophilic in nature [44,45,46]. Using hydro-organic solvents maximizes the potential of extracting canola phenols both qualitatively and quantitively. We employed various solvents with a wide range of polarities in order to comprehensively explore canola meal bioactive constituents. While hydro-alcoholic solvents are the most commonly used extraction solvent for plant phenols [47,48], hydro-acetone solvents showed superior quantitative properties [2,35]. Chloroform and hexane are immiscible with water, while n-butanol has limited water miscibility. Their mixtures with water form interesting two-phase extraction systems that have been frequently reported in the literature [49,50]. Chemical characterization of the extracts has been performed [35]. The prepared extracts were freeze-dried and referred to as acetone extract (AE), methanol (ME), butanol (BE), ethanol (EE), hexane (HE), chloroform (CE), and water (WE). All freeze-dried extracts were then reconstituted in 50% methanol and filtered through a syringe filter (0.22 μm).

### 4.3. Measurement of Total Phenolic Content

Total phenolic content (TPC) was determined using the Folin–Ciocalteu reagent, as previously described [2]. A calibration curve was produced using a range of gallic acid concentrations (0.1, 0.2, 0.3, 0.4, 0.5, and 0.6 mg/mL) in 50% aqueous methanol. A 100 µL aliquot of each standard or sample was added to a 10 mL volumetric flask, containing 7 mL of ultra-pure water (UPW) and then mixed with 500 µL of Folin–Ciocalteu reagent and 1.5 mL of 20% sodium carbonate. Following incubation for 1 h at room temperature, the absorbance was measured at 760 nm using a Cary 50 spectrophotometer (Varian, Victoria, Australia), with software Cary WinUV version 3, (Varian, Victoria, Australia), and results were expressed as milligrams of gallic acid equivalents (GAE) per gram dry weight (mg GAE/g DW).

### 4.4. Cell Culture and Adipogenic Differentiation

C3H10T1/2 cells were cultured in DMEM, containing 10% FBS, 1% penicillin-streptomycin, and 1% L-glutamine and incubated at 37 °C in a humidified incubator with 5% CO_2_. Media was replaced every 3 days. At 75% confluency, cells were trypsinized using 0.25% trypsin-EDTA solution for 2–5 min and then re-suspended in DMEM cell culture medium in a 1:10 ratio. Cell viability was assessed using the CellTiter 96® AQueous non-radioactive cell proliferation assay, according to the manufacturer’s protocol, with C3H10T1/2 cells plated at a density of 5000 cells/well in a 96-microplate cultured first with 0.1% to 0.3% DMSO with DMEM. Then, depending on the solubility of each extract, samples were used at a percentage of 1% to 3% with a suitable DMSO percentage. DMEM was used as a negative control. Following incubation for 24 h at 37 °C, absorbance was measured at 570 nm using a FLUOstar omega UV–vis spectrophotometer (BMG Labtech, Offenburg, Germany).

For adipogenic differentiation, C3H10T1/2 cells were cultured to 70% confluency and then exposed to adipogenic differentiation media (ADM) consisting of DMEM with 0.5 μM rosiglitazone, 10 μM insulin, 0.25 μM indomethacin, 1 μM dexamethasone, and 0.5 mM 3-isobutyl -1-methyxanthine (IBMX). ADM was replaced every 48 h, for a seven-day period before the cells were examined by Oil Red O staining.

### 4.5. Oil-Red O Staining Staining and Quantification of Intracellular Lipid Droplets

Oil Red O staining was performed according to the procedure described previously [51]. Briefly, C3H10T1/2 cells were treated with ADM containing 2 mg/mL of AE, ME, HE, CE, and WE or 1.5 mg/mL of BE and EE extracts for seven days. Cells were rinsed briefly with PBS, fixed, and air-dried before staining, then rinsed carefully with PBS, and viewed under an inverted microscope (Nikon Eclipse Ti–U inverted, Japan).

### 4.6. Immunofluorescence Staining of PPARγ

Cells were incubated with canola extracts (as detailed above) in ADM for seven days, after this period cells were fixed with 3% paraformaldehyde (PFA), rinsed with phosphate buffer saline (PBS), then treated with 0.1% Triton X-100 for 7 min at room temperature (RT), and rinsed again with PBS. The cells were then incubated for 30 min in blocking buffer prepared by mixing 5% goat serum (Gibco^®^, Eggenstein, Germany) in PBS. The cells were then incubated with anti-PPARγ (81B8) rabbit monoclonal antibody (1:50) Cell Signalling Technology (**Danvers**, **MA, USA**) for one hour at ambient temperature, then washed gently with PBS and incubated in the dark with anti-rabbit IgG (Fab 2)–Alexa Fluor^®^ 488 (1:100; Cell Signalling Technology) for one hour. Finally, they were counterstained with 4’,6-diamidino-2-phenylindole (DAPI) counterstain. Cells were observed using an A1R^+^/A1^+^confocal laser microscope system (Nikon, NY, USA).

### 4.7. Quantitative PCR (qPCR) of PPARγ Gene Expression

Total RNA was isolated from treated and un-treated C3H10T1/2 cells using the Aurum™ total RNA kit (Bio-Rad), following the manufacturer’s protocol. The concentration and quality of RNA were measured using a Nanodrop 2000’analyser (Thermo Scientific Ltd, Melbourne, Australia). The purity of the RNA samples was measured and an absorbance ratio at A260/A280 of 1.84 was achieved.

Complementary DNA (cDNA) was synthesized using the iScript Advance cDNA synthesis kit for RT-qPCR (Bio-Rad) according to the manufacturer’s protocol. The reverse transcription reaction was incubated in a thermocycler using the amplification cycles at 25 °C, 5 min; 42 °C, 30 min; 85 °C, 5 min, and 4 °C, 5 min. The amplification of the synthesized cDNA was performed by PCR at a final concentration of 300 ng/20 μL, using 12.5μL 2× GoTaq buffer, with 0.5 μL (1 μM) of forward and reverse primers specific for PPARγ and β-actin, the housekeeping gene.

The PCR primers for PPARγ1 were designed based on murine PPARγ 1 (accession number: NM_011146.2) [52]. PPARγ forward 5’ TTTTCAAGGGTGCC AGTTTC, reverse 5’AATCCTTGGCCCTCTGAGAT; β-actin with forward 5’CACCCGCGAGTACAACCTTC, reverse 5’CCCATACCCACCATCACACC. The PCR product sizes were 197 base pairs (bp) (PPARγ) and 207 bp (β-actin). Amplified products were separated by electrophoresis on 2% agarose gel and visualized with ethidium bromide. The gel images were analyzed and captured using the molecular imager gel doc XR+ system (Bio-Rad, NSW, Australia).

For real-time PCR amplification, a total volume of 20 μL reaction was prepared using 2× Soso fast mix (BioRad): 10 μL 2× Soso fast mix, 0.1 μL (at a final concentration of 0.2 μM) of forward and reverse primers, 1 μL of cDNA template (600 ng), with a total reaction volume of 20 μL with DNase-free water. The qPCR reactions were carried out using a ‘C1000 thermal cycler with real-time system CFX96’ (Bio-Rad) using the following parameters: initially at 95 °C, 3 min; followed by 95 °C, 1 min; 5 9 °C, 1 min; and repeated 39 times.

### 4.8. Pancreatic Lipase Inhibition

Inhibition of pancreatic lipase (PL) was determined as described [53]. CMEs were dissolved in Tris-HCl buffer (13 mM) containing 50 mM NaCl and 1.3 mM CaCl_2_. The reaction mixture was prepared in a 96-well microplate and included with 25 μL of each sample 50 μL of substrate [Orlistat] (0.1 mM), and 25 μL of PL enzyme (50 U/mL) and incubated at 25 °C for 30 min and the reaction was stopped with the addition of 100 μL of sodium citrate (100 mM, pH 4.2).

The relative fluorescence intensity was measured using a Cary eclipse fluorescence spectrophotometer (Varian, Inc, Victoria, Australia) at an excitation wavelength of 355 nm and an emission wavelength of 460 nm. The results were expressed as an IC_50_ value, obtained via a least square regression line of the logarithm of the amount of samples against the pancreatic lipase activity (%).

### 4.9. Chromatographic Characterization of Canola Extracts

For the online ABTS with HPLC-DAD and liquid chromatography-mass spectroscopy (LC-MS), the following standards were used: naringenin, gallic acid, catechin hydrate, caffeic acid, chlorogenic acid, ferulic acid, rutin, luteolin, sinapic acid, *trans*-cinnamic acid, 4-hydroxybenzoic acid, phenylisothiocyanate, quercetin, ellagic acid, tyramine, pyrogallol, and vanillic acid, which were purchased from Sigma Aldrich (Sydney, Australia). Epicatechin and kaempferol were obtained from Extrasynthese (Genay, France).

#### 4.9.1. Online High-Performance Liquid Chromatography with Diode Array Detector Coupled with ABTS Scavenging Activity (HPLC-DAD-Online ABTS).

All CME at 1.5 mg/mL concentration in 50 % aqueous methanol were filtered through a 0.22 µm syringe filter, following vortex/sonication before being analyzed by HPLC. All conditions were maintained as described by Obied et al. [2] with minor modifications. Each sample, blanks (50% aqueous methanol), and standards were analyzed for qualitative control and identification purposes.

Online ABTS with HPLC-DAD was achieved on a Varian Prostar 240 solvent delivery system connected with a Varian Prostar 410 autosampler. In addition, the outflow from HPLC-DAD was attached to a reaction coil (PEEK; 3.4 m × 0.178 mm, maintained at 37 °C) joined to a Perkin–Elmer series 10 HPLC pump (Varian 2401 pump). Changes in ABTS**^•+^** absorbance were measured at 414 nm using a Varian 9050 UV–vis detector. Data analysis was performed using the Star chromatography workstation version 6.41 2004 (Varian, Inc., CA, USA).

#### 4.9.2. High-Performance Liquid Chromatography-Diode Array Detection--Tandem Mass (HPLC-DAD-MS/MS).

For the HPLC-DAD-MS/MS, all required conditions were maintained as described by Obied et al. [2] with minor modifications. The total run time was 70 min including the MS procedure, and was performed in both the negative and positive ion mode (*m/z* 100−1200). Results were analyzed using an Agilent Mass Hunter workstation version B.01.04 2008 (Agilent Technologies, Waldbronn, Germany).

For quantitative determination, each extract was analyzed in triplicate at 280 nm and the mean reported. Sinapic acid (0.0625 to 1100 μg/mL) was used as the standard to generate a calibration curve for quantification (*R^2^* = 0.9935) and concentrations expressed as milligram of sinapic acid equivalent per gram of dry weight (mgSAE/g DW).

### 4.10. Statistical Analysis

Experiments were performed in triplicate, and results are presented as the mean ± standard deviation (SD). All results were analyzed using Graph pad prism 5, Microsoft Excel 2016, and one-way analysis of variance (ANOVA) using statistical analysis system (SAS^®^system) for Windows V8 (SAS institute, NC, USA). Comparison between sample means were calculated using the Duncan multiple range test at a 5% probability level (*p <* 0.05).

## 5. Conclusions

The most abundant phenols in CMEs are in AE, ME, and BE. These extracts demonstrated potent anti-adipogenic and anti-lipase activities. At the molecular level, a marked reduction in PPARγ mRNA expression was associated with AE and BE treatment of adipogenic differentiating cells. CME showed ABTS radical scavenging activity. AE has the highest content of TP and the most potent ABTS scavenging, anti-adipogenic, and PL inhibition. Sinapine and derivatives of sinapic acid, namely, kaempferol and ferroyl choline guiacyl, are the main contributors to CME antiradical activities. The results obtained in this study demonstrate the importance of solvent choice for the recovery of biophenols and in the observed pharmacological properties. Phenols are the main active constituents responsible for antioxidant and anti-adipogenic activity. Further research is required to isolate the main bioactive phenols, study the mechanism of action and find out if these activities are reproducible in vivo.

## Figures and Tables

**Table 1:** Major compounds identified in acetone extract as represented in Figure 2.

Peak No.	Identity	AE	RT	λ_max_	ABTS	ESI^+^	ESI^-^	MW
**1**	Sinapine	✓✓✓	15.7	237, 328	**++**	310	294, 663	310
**2**	Ferroyl choline(4-0-8’) guiacyl	✓✓	17.7	270b, 325b	**+++**	476	NI	476
**3**	Unknown	✘	24.6	260s, 280s, 290s	**++**	429	427	428
**4**	Ferroyl choline guiacyl isomer	✓	18.3	270b, 325b	**++**	476	NI	476
**5**	Kaempherol-sinapoyl-trihexoside	✓✓	26.3	268, 333	**+**	979	978	977
**6**	*Trans*-sinapic acid	✓✓	32.6	324	**++**	NI	223	224
**7**	Feruloyl choline (4-O-8’) guaiacyl-di-sinapoyl	✓✓	36.1	323	**++**	682	NI	682
**8**	Disinapoyl dihexoside	✓✓	38.6	230, 330	**++**	NI	753	754
**9**	Disinapoyl hexoside	✓✓	40.7	330	**++**	NI	591	592

AE, acetone extract; ✓ less; ✓✓, medium; ✓✓✓, high peak; ✘, no peak; RT, retention time; λ_max_, UV–vis spectra; ESI^−^, electrospray ionization peaks in negative mode; ESI^+^, electrospray ionization peaks in positive mode; MW, molecular weight; NI, did not ionize under ESI modes; b, broad peak; s, peak shoulder; ABTS (2,2’-azino-bis(3-ethylbenzothiazoline-6-sulfonic acid) representing ABTS scavenging activity in online assay (antioxidant); +, low ABTS scavenging activity; ++, good ABTS scavenging activity; +++, high ABTS scavenging activity.
